# Electrodeposition of Cobalt Oxides on Carbon Nanotubes for Sensitive Bromhexine Sensing

**DOI:** 10.3390/molecules27134078

**Published:** 2022-06-24

**Authors:** Sireerat Lisnund, Vincent Blay, Pratchaya Muamkhunthod, Kittiya Thunyanon, Jaruwan Pansalee, Jirawan Monkrathok, Pachara Maneechote, Kantapat Chansaenpak, Piyanut Pinyou

**Affiliations:** 1 Department of Applied Chemistry, Faculty of Science and Liberal Arts, Rajamangala University of Technology Isan, Nakhon Ratchasima 30000, Thailand; mpratchaya28@gmail.com (P.M.); kittiya.28082556@gmail.com (K.T.); 2 Department of Microbiology and Environmental Toxicology, University of California Santa Cruz, Santa Cruz, CA 95064, USA; vroger@ucsc.edu; 3 School of Chemistry, Institute of Science, Suranaree University of Technology, Nakhon Ratchasima 30000, Thailand; jaruwon.pa2565@gmail.com; 4 Institute of Research and Development, Suranaree University of Technology, Nakhon Ratchasima 30000, Thailand; jirawanp5414@gmail.com (J.M.); pachara.ma@sut.ac.th (P.M.); 5 National Nanotechnology Center, National Science and Technology Development Agency, Thailand Science Park, Pathum Thani 12120, Thailand; kantapat.cha@nanotec.or.th

**Keywords:** bromhexine hydrochloride, carbon nanotubes, cobalt oxide, amperometric sensor

## Abstract

We develop an electrochemical sensor for the determination of bromhexine hydrochloride (BHC), a widely use mucolytic drug. The sensor is prepared by electrodeposition of cobalt oxides (CoO_x_) on a glassy carbon electrode modified with carboxylated single-walled carbon nanotubes (SWCNT). A synergistic effect between CoO_x_ and SWCNT is observed, leading to a significant improvement in the BHC electrooxidation current. Based on cyclic voltammetry studies at varying scan rates, we conclude that the electrochemical oxidation of BHC is under mixed diffusion–adsorption control. The proposed sensor allows the amperometric determination of BHC in a linear range of 10–500 µM with a low applied voltage of 0.75 V. The designed sensor provides reproducible measurements, is not affected by common interfering substances, and shows excellent performance for the analysis of BHC in pharmaceutical preparations.

## 1. Introduction

Bromhexine hydrochloride (BHC or 2-amino-3,5-dibromo-*N*-cyclohexyl-*N*-methylbenzylamine hydrochloride) is a small-molecule mucolytic drug [1]. BHC reduces the viscosity of mucus and enhances lysosomal activity, resulting in the hydrolysis of mucopolysaccharides. BHC is thus used to facilitate mucus clearance in different respiratory disorders, including the treatment of bronchiectasis and emphysema in chronic obstructive pulmonary disorder (COPD) [2]. Recently, Ansarin et al. reported the oral administration of BHC in the early treatment of COVID-19 patients and showed positive outcomes, including reduced ICU transfer, intubation, and mortality rates [3]. However, there are also risks involved with BHC use. For example, patients with a history of gastric ulcers should avoid BHC because it can deteriorate mucosal barriers in the gastrointestinal tract. Moreover, high doses of BHC can cause nausea, rash, diarrhea, and other adverse effects. Thus, the accurate quantification of BHC in pharmaceutical products and clinical samples is critical [4]. Several analytical techniques have been employed for BHC analysis, including high-performance liquid chromatography (HPLC) [5], spectrophotometry [6], electrochemiluminescence [7], and potentiometric titration [8]. These methods require sophisticated and expensive instruments, which are often not available in low-resource areas. The electrochemical analysis of BHC seems like a promising alternative, as it could enable portable instruments with reasonable sensitivity [9]. The electrochemical oxidation mechanism of BHC has been elucidated [10], and it seems suitable for the development of an electrochemical sensor.

A major obstacle in electrochemical sensing is the need for large overpotentials to detect an analyte and the consequent electrode fouling, which can deteriorate its performance and stability [11]. Many surface modifications have been proposed to reduce fouling accretion on electrodes [12]; a promising approach is based on coating the electrode surface with metal-oxide nanoparticles, which reduces the overpotentials required [13]. Cobalt oxide nanoparticles are particularly interesting due to their unique properties, including large surface area, high dispersion (fraction of atoms on the surface), and great chemical stability [14]. Electrodes modified with cobalt oxide nanoparticles showed excellent electrocatalytic activity on glucose [15], arsenic(III) [16], nitrite [17], H_2_O_2_ [18]_,_ and *p*-nitrophenol [19]. Several preparation methods for cobalt oxide nanoparticles have been reported based on hydrothermal synthesis [20], precipitation [21], electrodeposition [22,23], as well as synthesis using leaf extracts [24]. Electrodeposition enables the formation of cobalt oxide nanoparticles in situ on an electrode in a fast and controlled manner [25]. Moreover, the electrodeposition of cobalt oxides directly onto an electrode surface is generally a simpler and more controllable method [26]. Specific advantages include controlling the amount deposited through the electricity supplied over time as well as enabling the fabrication of heterojunctions between oxides and sulfide materials, in which a strong adsorption between two phases is established through electrodeposition [27].

Another strategy to further enhance electrochemical sensors is to modify the electrodes with carbon-based nanomaterials, such as multi-walled carbon nanotubes (MWCNTs), single-walled carbon nanotubes (SWCNTs), graphene, or carbon nanofibers [28]. Thanks to their large electroactive surface area and excellent electrical conductivity, these nanomaterials often help decrease the overpotentials and improve the performance of electrochemical sensors [29,30]. Carboxylated SWCNTs are particularly attractive [31] because the carboxyl groups minimize the agglomeration of CNTs and allows their dispersion in water [32].

In this work, we proposed an amperometric sensor for BHC determination in pharmaceutical preparations. Carboxylated SWCNTs were casted on the surface of glassy carbon electrode (GCE) followed by the electrodeposition of cobalt oxide nanoparticles (CoO_x_ NPs) by cyclic voltammetry. The prepared electrode was characterized using voltammetry, electrochemical impedance spectroscopy, and electron microscopy. The electrode was then evaluated as a sensor for BHC; it demonstrated a reduction in overpotentials required for BHC oxidation and a significant increase in sensitivity compared to an unmodified electrode. The designed sensor was successfully used for the determination of BHC in pharmaceutical preparations and demonstrated the advantages of combining carboxylated SWCNTs with cobalt oxide nanoparticles.

## 2. Results and Discussion

### 2.1. Electrodeposition of CoO_x_NPs on SWCNTs/GCE

**Figure 1:**
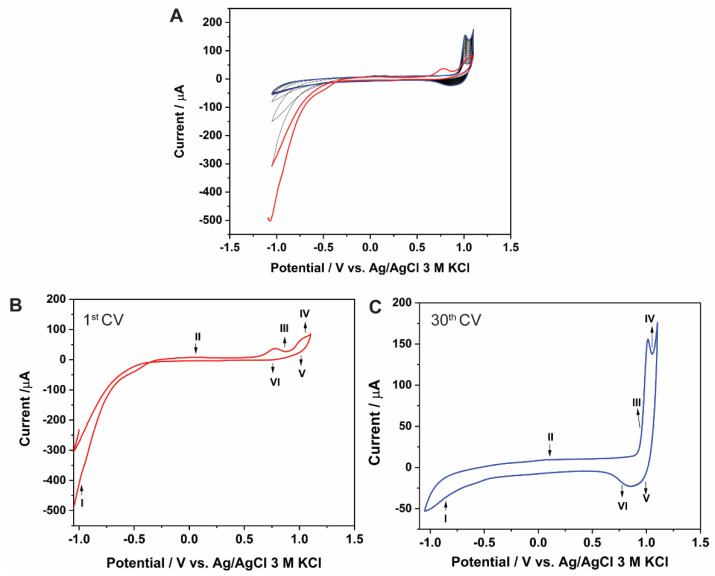
(**A**) Cyclic voltammograms for the SWCNTs/GCE recorded in 0.1 M CoCl_2_ solution with 0.1 M phosphate buffer pH 7 at a scan rate of 50 mV/s. (**B**) 1st CV. (**C**) 30th CV.

The electrodeposition of CoO_x_NPs on a SWCNTs-modified GCE was adapted from Salimi et al. [16,33]. The SWCNT/GCE was subjected to 30 cyclic voltammetry cycles in the range −1.1 to 1.1 V in 0.1 M phosphate buffer pH 7.0 containing 0.1 M CoCl_2_. A variety of peaks can be observed in the cyclic voltammogram (CV) (Figure 1A), which are described next.

Figure 1B highlights the first CV scan. The cathodic peak (peak I) at −1.05 V corresponds to the reaction:

Co^2+^ + 2e^−^ ⇌ Co (s) (1)

The cathodic peak current of peak I decreases with the number of scan cycles, indicating that the Co(II) ions are reduced to metallic cobalt. A small anodic peak (peak II) is visible at 0.1 V in the reverse scan, which is associated with the dissolution of the cobalt layer on the electrode surface (reverse of Equation (1)) [34,35].

The anodic peak at 0.9 V (peak III) is associated with Co_3_O_4_ film formation by reaction (2).

3Co(OH)_2_ + 2OH^−^ → Co_3_O_4_ + 4H_2_O + 2e^−^ (2)

The peak at the high oxidation potential of ca. 1.1 V (peak IV) corresponds to the oxidation of Co(OH)_2_ or Co_3_O_4_ into CoOOH, indicated by Equations (3) and (4) [29].

Co(OH)_2_ + OH^−^ → CoOOH + 2H_2_O + e^−^ (3)

Co_3_O_4_ + H_2_O + OH^−^ → 3CoOOH + e^−^ (4)

The reductions at 1.1 V and 0.75 V (peak V and peak VI in Figure 1C) are related to the conversion of CoOOH into Co(OH)_2_ or Co_3_O_4_ (reverse of Equations (3) and (4)). As a result, the electrodeposited CoO_x_ consist of different cobalt-containing phases, including Co_3_O_4_ and CoOOH.

### 2.2. Surface Morphology of the Electrodes

**Figure 2:**
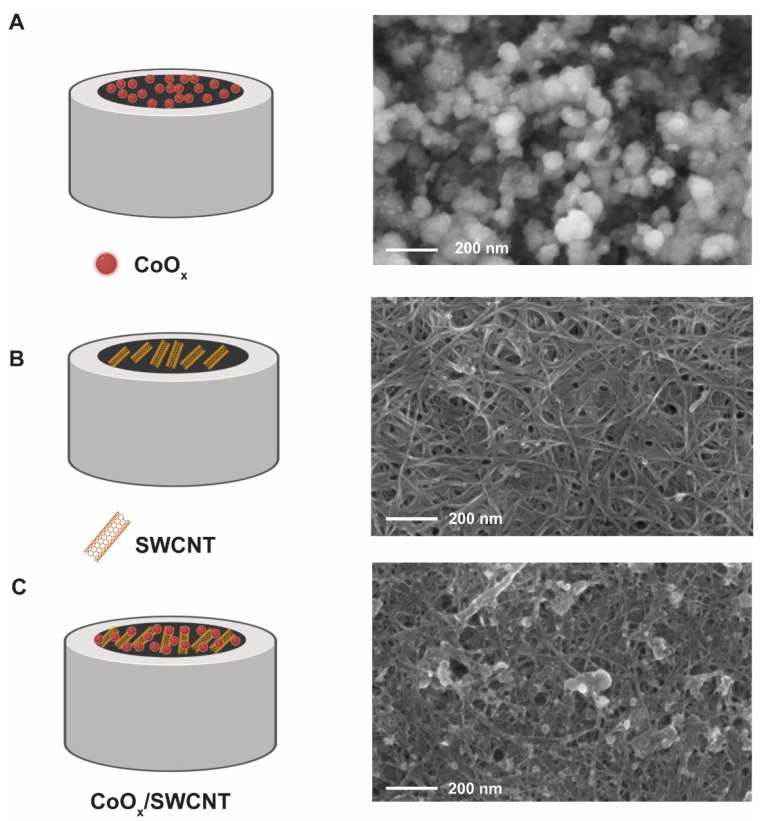
Diagram of the electrode components and FE-SEM images at 50,000× of (**A**) CoO_x_/GCE (**B**) SWCNT/GCE (**C**) CoO_x_/SWCNT/GCE. Diagrams created with Biorender.com.

The surface of the electrodes upon different modifications was investigated by SEM. Firstly, a CoO_x_ film was directly deposited on the GCE surface, leading to discrete CoO_x_NPs particles of different sizes (Figure 2A). The formation of such heterogeneous, discontinuous film agrees with the observations by Spataru et al. [33]. Secondly, the GCE surface was modified with SWCNTs by drop-casting. In this case, a three-dimensional network of SWCNTs with high porosity and large surface area can be clearly observed in Figure 2B. Thirdly, when the CoO_x_ film was electrodeposited on the SWCNT/GCE, we observe the formation of discrete CoO_x_ particles on the SWCNTs mesh (Figure 2C). In this case, CoO_x_NPs are formed upon adsorption of Co(II) on the SWCNTs surface, enabling the heterogeneous nucleation of CoO_x_. The chemical composition of the CoO_x_/SWCNTs/GCE surface was investigated by energy-dispersive X-ray spectroscopy (EDX). The results are shown in Figure S1 and Table S1 and evidence the presence of C, O, and Co, confirming the deposition of CoO_x_ on the layer of SWCNT. Some elements such as Na, P, and Cl are detected at low levels (less than 1%) and likely originate from the electrodeposition buffer.

### 2.3. Electrochemical Impedance Spectroscopy

The electrical conductivity and charge transfer resistance (R_ct_) are essential properties of an electrochemical sensor. Electrochemical impedance spectroscopy (EIS) is a useful technique that can evaluate these properties and how they are affected by different modifications.

**Figure 3:**
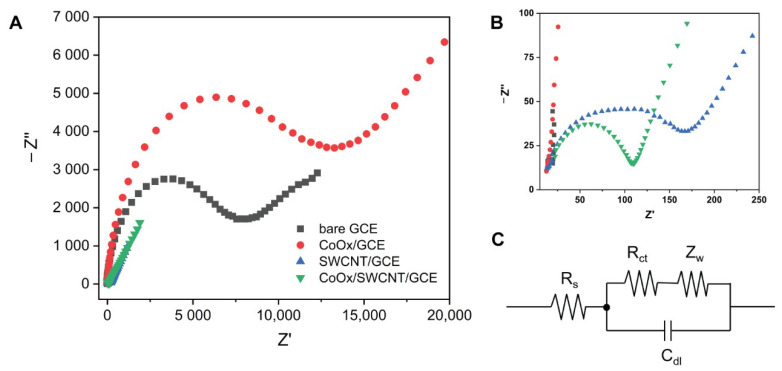
(**A**) Nyquist plots for the redox probe couple 5 mM Fe(CN)_6_^4−^/^3−^ over electrodes with different modifications in 0.1 M KCl. (**B**) Nyquist plots at high frequency range. (**C**) Equivalent Randles circuit used for fitting the Nyquist plots; R_s_ is the electrolyte resistance; R_ct_ is the charge transfer resistance; C_dl_ is the double layer capacitance; Z_w_ is the Warburg impedance.

The Nyquist plots of the electrodes in this work are shown in Figure 3. The unmodified GCE gives a R_ct_ value of ca. 6 kΩ (Table 1). For the CoO_x_/GCE, the R_ct_ observed shows the highest R_ct_ value among all the electrodes investigated—ca. 9.7 kΩ. This is attributed to the low conductivity of CoO_x_ nanoparticles on the GCE surface, which hinders the electron transfer from the redox couple of Fe(CN)_6_^4−^/^3−^. The SWCNT/GCE displayed a small R_ct_ value of 120 Ω thanks to the excellent conductivity and high surface area provided by the CNTs. The CoO_x_/SWCNT/GCE design shows the lowest R_ct_ value of only 70 Ω. The EIS results thus demonstrate that a combination of CoO_x_ nanoparticles and SWCNT drastically accelerates the electron transfer. Such low resistance is enabled by the synergistic effect between SWCNT and CoO_x_ nanoparticles [15]. The electroactive surface area of the GCE with different modifications calculated from cyclic voltammetry in the redox probe solution of 5 mM K_3_[(Fe(CN)_6_] containing 0.1 M KCl are presented in Figure S2 and Table S2.

### 2.4. Electrochemical Behavior of BHC on CoO_x_/SWCNT/GCE

**Figure 4:**
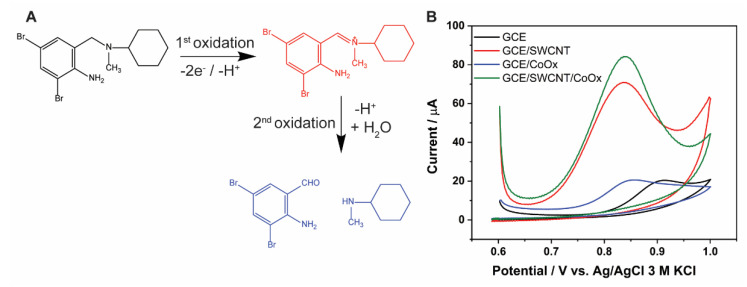
(**A**) Schematic diagram of BHC electrochemical oxidation (**B**) Cyclic voltammograms of 1 mM BHC in 0.1 M phosphate buffer pH 5.5 over bare GCE, CoO_x_/GCE, SWCNT/GCE and CoO_x_/SWCNT/GCE at a scan rate of 100 mV/s.

BHC can be electrochemically oxidized (Figure 4A) and a mechanism has been previously proposed [10]. The reaction involves two electrons and two protons with two sequential oxidation steps. In this work, the detection of BHC was based on the first oxidation step. The second oxidation step yields a lower signal and was not used. We investigated the electrochemical behavior of BHC by cyclic voltammetry using electrodes with different modifications in a phosphate buffer pH 5.5.

The CVs of the electrodes in the presence of 1 mM BHC are shown in Figure 4B. The results clearly show the impact of the electrode modifications on the BHC oxidation peak. The unmodified GCE showed the highest overpotential for BHC oxidation at 0.9 V. The oxidation peak for CoO_x_/GCE occurred at a lower overpotential of ca. 0.85 V, with a peak current similar to that of the unmodified GCE. The lower potential value might be due to a favorable electrostatic interaction between the positively charged BHC and the oxygen species in CoO_x_.

On SWCNT/GCE, BHC oxidation was clearly observed at a peak potential of ca. 0.83 V, with a drastic increase in the anodic current up to 60 µA. When CoO_x_ nanoparticles were electrochemically deposited on the surface of SWCNT/GCE, the BHC oxidation peak maintained this low overpotential and showed the highest peak current of all the electrodes studied, ca. 75 µA. This result is consistent with the EIS and confirms that the co-localization of SWCNT and CoO_x_ nanoparticles on the GCE surface enables a favorable electron transfer in BHC oxidation. Therefore, the CoO_x_/SWCNT/GCE electrode design was chosen for further study.

### 2.5. Effect of pH

**Figure 5:**
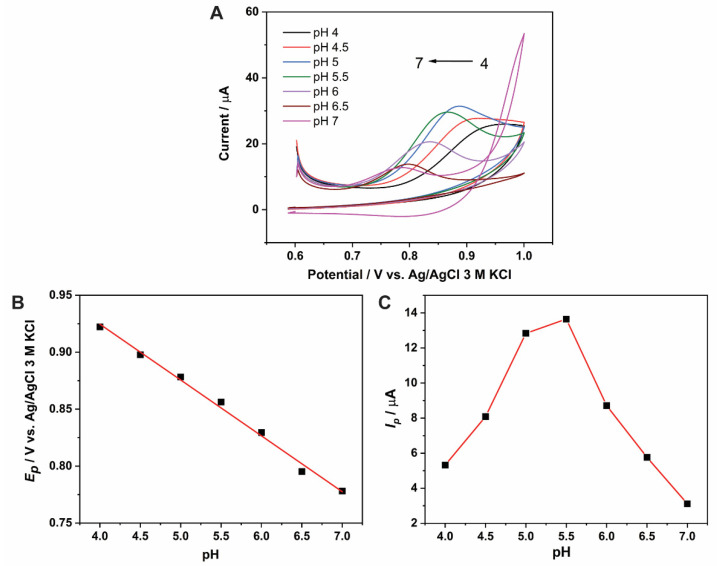
(**A**) Cyclic voltammograms of 1 mM BMC on CoO_x_/SWCNT/GCE in phosphate buffer at varying pH from 4 to 7; scan rate: 100 mV/s: potential range: 0.6–1.0 V. (**B**) Plot of anodic peak potential vs. pH. (**C**) Plot of anodic peak current vs. pH.

The two-step oxidation of BHC produces two protons, two electrons, and their respective products, as shown in Figure 4A. Since BCH oxidation involves the loss of protons, the pH of the electrolyte solution plays a crucial role. The CVs of 0.1 M BHC in 0.1 M phosphate buffers with pH ranging from 4 to 7 are shown in Figure 5A. The anodic peak potential decreases as the pH of the solution increases (Figure 5B). A linear decline of the BHC oxidation potential with the pH is obtained according to the equation E_pa_ (V) = −0.049pH + 1.1206 (R^2^ = 0.9941). The slope of this line (−0.049) is slightly higher than the theoretical Nernst value of −0.059, indicating that the first electrooxidation of BHC involves a two-electron, one-proton process [10].

Although the second oxidation peak might be observed at a potentials higher than 1 V [10], we focused on the first oxidation peak given its stronger signal. The anodic peak currents are plotted against the pH in Figure 5C. An increase in peak current was observed between pH 4 and 5.5, and a decrease was observed at pH values higher than 5.5. This behavior may be attributed to the precipitation of BHC at a high pH [10], resulting in a decrease in the anodic oxidation current. A pH value of 5.5 was thus selected for subsequent studies.

### 2.6. Effect of Scan Rate

**Figure 6:**
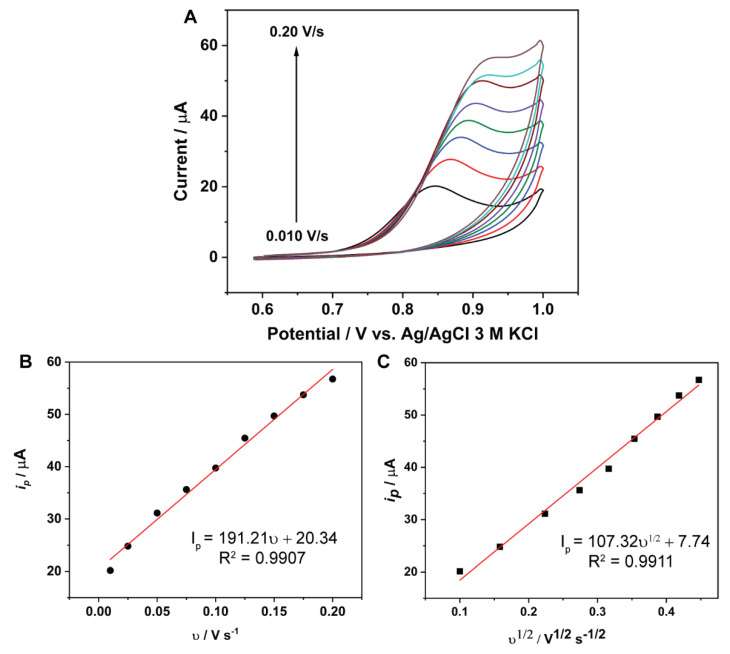
(**A**) Cyclic voltammograms of 1 mM BHC at the CoO_x_/SWCNT/GCE in phosphate buffer pH 5.5 at different scan rates from 0.010 to 0.20 V/s; potential range: 0.6−1.0 V. (**B**) a plot of anodic peak potential vs. scan rate. (**C**) A plot of anodic peak current vs. square root scan rate.

We investigated the effect of the scan rate on the electrooxidation of BHC. Cyclic voltammetry was carried out with CoO_x_/SWCNT/GCE in phosphate buffer pH 5.5 and 1 mM BHC at scan rates in the range 10–200 mV/s. The results, in Figure 6A, show an increase in the anodic peak current with the applied scan rate. Moreover, the anodic peak potential of BHC also increased with the scan rate. We believe that this behavior is due to the irreversible oxidation of BHC on the electrode surface. The anodic peak current values (*I_p_*) were plotted against the scan rate (*υ*) and square root scan rate (*υ*^1/2^), shown in Figure 6B,C, respectively. The plots show a reasonable linearity between the peak current and *υ* or *υ*^1/2^. This indicates that both adsorption and diffusion rates of BHC contribute similarly to the observed kinetics of the process, rather than one phenomenon dominating the kinetics.

### 2.7. Analytical Characteristics of the Proposed Electrode

**Figure 7:**
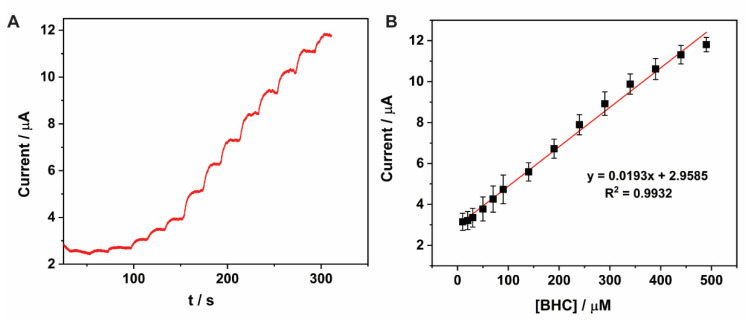
(**A**) Chronoamperometric response after successive additions of BHC with the resulting concentration ranging from 10 to 500 µM. Measurements were carried out with CoO_x_/SWCNT/GCE in phosphate buffer pH 5.5; applied potential = 0.75 V vs. Ag/AgCl 3 M KCl, stirring = 300 rpm. (**B**) Calibration curve for BHC determination (*n* = 3).

The electrode design CoO_x_/SWCNT/GCE was studied as an amperometric BHC sensor. A constant potential of 0.75 V was chosen for carrying out a BHC calibration. This value was selected to attain a high current from the BHC oxidation while avoiding the electrode fouling that can take place over time at higher operating potentials [36]. The current response was monitored as BHC was added to a phosphate buffer pH 5.5. The electrolyte was continuously stirred at 300 rpm, which accelerates the mass transport by forced convection. A pronounced increase in amperometric response was observed as the BHC concentration increased (Figure 7A). A steady-state current was attained in less than 20 s after each addition, showing that the CoO_x_/SWCNT/GCE had a fast response time. A calibration curve for BHC amperometric sensing was built with concentrations ranging from 10 to 500 µM. The linear equation obtained is *I_p_* (µA) = 0.0193*c* (µM) + 2.9585 (R^2^ = 0.9937), which is plotted in Figure 7B. At BHC concentrations above 500 µM, a deviation from linearity can be observed, which may be due to the adsorption of the reaction products generated (Figure 4A), resulting in fouling.

The limit of detection (LOD) of the BHC sensor was 8.1 µM, estimated from 3*σ*/*m*, where *σ* is the standard deviation of a blank and *m* is the slope of the BHC calibration graph. Table 2 compares the performance of BHC sensors reported in different studies. Our CoO_x_/SWCNT/GCE design shows a wider linear range for BHC determination than previous sensors, enabling its use for the analysis of pharmaceutical preparations with higher BHC levels. Moreover, the detection of BHC based on amperometry with forced convection in this work offers shorter analysis times than adsorption-differential pulse-voltammetry (DPV requires several minutes for the analyte to adsorb on the electrode surface).

### 2.8. Reproducibility, Repeatability and Interference Studies

The reproducibility of the CoO_x_/SWCNT/GCE sensor was examined by performing amperometric measurements of 300 µM BHC with five independently prepared electrodes. The current response obtained shows a relative standard deviation (RSD) of 9.60%, indicating that the proposed sensor offers reproducible performance for BHC analysis. Moreover, the repeatability of the modified electrode was evaluated by measuring the current response from a 300 µM BHC solution five times with one single electrode. The results show an excellent repeatability, with a RSD of 3.96%.

The effect of potential interferents on BHC detection was investigated by measuring the amperometric response of 50 µM BHC in phosphate buffer pH 5.5 in the presence of different substances, including glucose, sucrose, ribose, caffeine, NH_4_Cl, MgCl_2_, and KNO_3_. Although these substances were introduced at individual concentrations 100 times higher (30 mM) than that of BHC, they had no significant effect on the BHC current response (less than 10%), as shown in Table S3. These results confirm that the proposed sensor shows high selectivity towards BHC over these potential interferents.

### 2.9. Real Sample Analysis

The proposed electrochemical sensor was used to determine the BHC concentration in pharmaceutical preparations. Prior to BHC analysis, a commercial tablet was ground and diluted with phosphate buffer pH 5.5, while the liquid formulation was diluted at a ratio 1:40 with the same buffer. The BHC concentration was determined by the standard addition method. The results obtained with the CoO_x_/SWCNT/GCE amperometric sensor are shown in Table 3. The BHC contents determined in the tablet and liquid formulation are 8.26 mg/tablet and 4.55 mg/5 mL, respectively, while the nominal values are 8 mg/tablet and 4 mg/5 mL. The %recovery values are close to 100% and the variation between repeated measurements is very low, highlighting the excellent performance of the CoO_x_/SWCNT/GCE sensor.

## 3. Materials and Methods

### 3.1. Reagents and Materials

Bromhexine hydrochloride and cobalt chloride were obtained from ChemPUR (Karlsruhe, Germany). Single-walled carbon nanotubes functionalized with 3–6% -COOH groups (P3-SWCNT) was purchased from Carbon Solutions, Inc. (Riverside, CA, USA). Disodium hydrogen phosphate, potassium dihydrogen phosphate, and potassium chloride were obtained from QrëC (Auckland, New Zealand). All chemicals used in this work were of analytical grade. All aqueous solutions were prepared with deionized (DI) water.

### 3.2. Apparatus

All voltammetric experiments were performed at room temperature using a three-electrode setup equipped with a potentiostat (an Autolab PGSTAT204 with Nova2.1 software package, Utrecht, The Netherlands). The three-electrode setup for the electrochemical measurements consisted of a Ag/AgCl 3 M KCl as the reference electrode, a Pt sheet (1 × 1 cm) as the counter electrode, and a glassy carbon electrode (GCE) as the working electrode (WE). Electrochemical Impedance Spectroscopy was conducted using a PalmSens 4 EIS potentiostat/galvanostat controlled by PSTrace 5.8 software (PalmSens, Houten, The Netherlands). The same reference and counter electrodes were used for the impedance measurements of the GCE and the modified GCE. The pH values of the buffer solution were determined using a calibrated pH meter (Mettler Toledo, Columbus, OH, USA). The surface morphologies of the GCE electrode surface with different modifications were investigated using a field-emission scanning electron microscope (Zeiss AURIGA FE-SEM/FIB/EDX, Carl Zeiss Microscopy GmbH, Jena, Germany) at 1 keV acceleration and 30 µm aperture.

### 3.3. Modification of the GCE

Prior to modification, the glassy carbon electrode (GCE, 3 mm diameter) surface was sequentially polished on chamois leather containing 5, 1, and 0.5 μm alpha-alumina (Al_2_O_3_) slurry. It was then sonicated in DI water for 10 min and dried in air. SWCNT were dispersed in ethanol (4 mg/mL) in an ultrasonication bath for 30 min. Then, 2 µL of the SWCNT suspension was drop-casted on the surface of the GCE and allowed to dry under an infrared lamp. The SWCNT/GCE was immersed in 0.1 M cobalt chloride in 0.1 M phosphate buffer pH 7. A cobalt oxide film (CoO_x_) was created by cyclic voltammetry with a potential range from −1.1 to 1.1 V over 30 cycles at a scan rate of 50 mV/s. The same technique without the SWCNT casting was used to prepare the CoO_x_/GCE.

### 3.4. Electrochemical Impedance Spectroscopy

EIS measurements were carried out in a solution containing 5 mM Fe(CN)_6_^4−^/**^3^**^−^ in 0.1 M KCl with an open-circuit potential of 215 mV, at frequencies ranging from 100 kHz to 50 mHz and an AC amplitude of 5 mV.

### 3.5. Preparation of Real Samples

For the bromhexine tablet, an accurately weighed portion (0.1210 g) of each homogenized tablet (BROMSTAR©, nominal content of 8 mg BHC/tablet) was dissolved with 10 mL DI water. For the liquid drug formulation (BISOZIN©, nominal content of 4 mg BHC per 5 mL), 250 μL of the sample was transferred to an electrochemical cell containing 10 mL of phosphate buffer pH 5.5. No pretreatment was used for any of the samples.

## 4. Conclusions

We propose a facile approach for fabricating an electrochemical sensor useful for the determination of BHC. A CoO_x_ film is deposited by cyclic voltammetry on a SWCNT-modified GCE. The presence of both SWCNT and CoO_x_ leads to an improvement in the current response from the electrochemical oxidation of BHC. After optimizing the measurement conditions, the proposed sensor exhibited a high sensitivity and a wide analytical range for the determination of BHC. Moreover, the sensor delivered reproducible analytical results and a good selectivity towards BHC oxidation. The application of the proposed sensor for BHC determination in pharmaceutical samples was also successfully demonstrated.

## Figures and Tables

**Table 1:** Comparison of the charge transfer resistance (R_ct_) estimated for the electrodes with different modifications.

Electrode	R_ct_ (Ω)
Bare GCE	5910
CoO_x_/GCE	9742
SWCNT/GCE	120
CoO_x_/SWCNT/GCE	70

**Table 2:** Comparison of electrochemical sensors reported for the determination of bromhexine hydrochloride.

Modified Electrode	Method	Linear Dynamic Range (µM)	LOD (µM)	Ref.
Poly(procaterol hydrochloride)/MWCNT/GCE	DPV	0.2–1.0 and 1.0–8.0	0.1	[4]
Glassy Carbon Electrode (GCE)	DPV	20–100	14	[10]
Ni-nanoparticles/MWCNT/Pt	SWV	5–230	3.0	[37]
Glassy carbon paste-flow injection	Amperometry	0.31–2.0	0.31	[38]
CoO_x_/SWCNT/GCE	Amperometry	10–500	8.1	This work

**Table 3:** Results of BHC analysis in pharmaceutical formulations by amperometric measurement with the CoO_x_/SWCNT/GCE under the optimized conditions.

Sample	Added (µM)	Found (µM)	%Recovery	%RSD ^1^
Tablet 1	-	50.09	-	2.34
	50	103.48	103.48	9.58
	100	152.14	101.37	5.54
Liquid formulation 2	-	55.10	-	6.08
	50	110.12	104.78	2.96
	100	171.15	110.34	1.33

^1^ Relative standard deviation of three measurements.

## Data Availability

Not applicable.

## Supplementary Materials

The following supporting information can be downloaded at: https://www.mdpi.com/article/10.3390/molecules27134078/s1, Figure S1: SEM-image of CoO_x_/SWCNT/GCE and its SEM-EDX spectrum of CoO_x_ particles on the SWCNT/GCE; Table S1: EDX analysis of the CoO_x_/SWCNT/GCE; Figure S2: Cyclic voltammograms of the GCE with different modifications in 5 mM K_3_[Fe(CN)_6_] containing 0.1 M KCl at the scan rate of 50 mV/s; Table S2: Comparison of the electroactive surface area for the electrode with different modifications estimated from CV in 5 mM K_3_[Fe(CN)_6_] containing 0.1 M KCl; Table S3: Effect of interfering substances on the current response for BHC determination (50 µM) using the CoO_x_/SWCNT/GCE.

## Sample Availability

Samples of the compounds are not available from the authors.
