# Supplementary material for: Electrodeposition of Cobalt Oxides on Carbon Nanotubes for Sensitive Bromhexine Sensing
Source: Molecules. 2022 Jun 24;27(13):4078. doi: 10.3390/molecules27134078 (PMC9268198; doi:10.3390/molecules27134078)
Supplement: Supplementary file 1 [file molecules-27-04078-s001.zip › molecules-1764581-supplementary.pdf]

Supplementary Material

Electrodeposition of Cobalt Oxides on Carbon Nanotubes for Sensitive Bromhexine Sensing

Sireerat Lisnund [1,*], Vincent Blay [2], Pratchaya Muamkhunthod [1], Kittiya Thunyanon [1], Jaruwan Pansalee [3], Jirawan Monkrathok [4], Pachara Maneechote [4], Kantapat Chansaenpak [5] and Piyanut Pinyou [3,*]

[1] Department of Applied Chemistry, Faculty of Science and Liberal Arts, Rajamangala University of Technology Isan, Nakhon Ratchasima 30000, Thailand; mpratchaya28@gmail.com (P.M.); kittiya.28082556@gmail.com (K.T.)

[2] Department of Microbiology and Environmental Toxicology, University of California Santa Cruz, Santa Cruz, CA 95064, USA; vroger@ucsc.edu

[3] School of Chemistry, Institute of Science, Suranaree University of Technology, Nakhon Ratchasima 30000, Thailand; jaruwon.pa2565@gmail.com

[4] Institute of Research and Development, Suranaree University of Technology, Nakhon Ratchasima 30000, Thailand; jirawanp5414@gmail.com (J.M.); pachara.ma@sut.ac.th (P.M.)

[5] National Nanotechnology Center, National Science and Technology Development Agency, Thailand Science Park, Pathum Thani 12120, Thailand; kantapat.cha@nanotec.or.th

* Correspondence: sireerat.in@rmuti.ac.th (S.L.); piyanutp@sut.ac.th (P.P.)

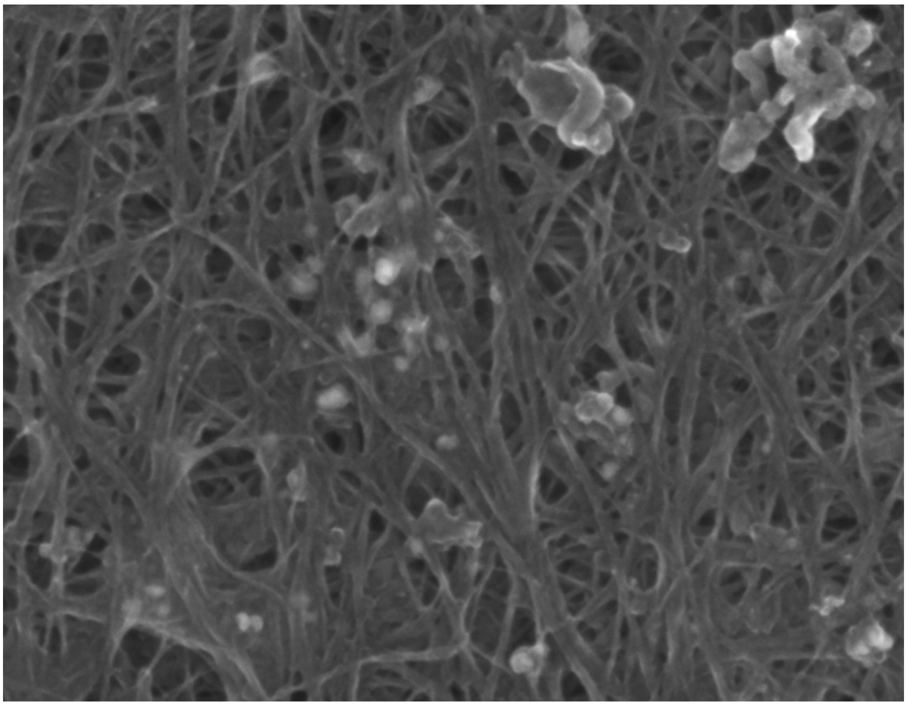

A

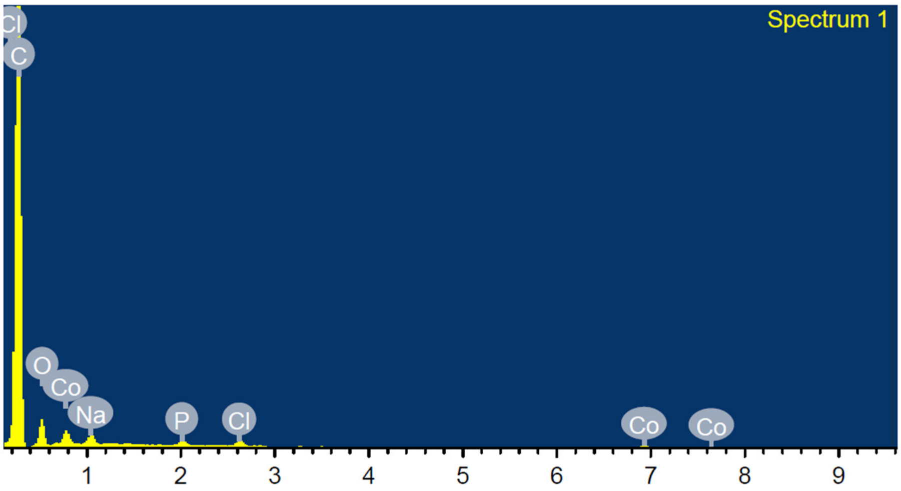

B

Figure S1. (**A**) SEM-image of CoO_x/SWCNT/GCE and (**B**) its SEM-EDX spectrum of CoO_x particles on the SWCNT/GCE.

Table S1. EDX analysis of the CoO_x/SWCNT/GCE .

Element	Series	%Weight	%Atomic
C	K	82.82	90.69
O	K	8.17	6.72
Na	K	0.90	0.52
P	K	0.61	0.26
Cl	K	0.95	0.35
Co	L	6.54	1.46

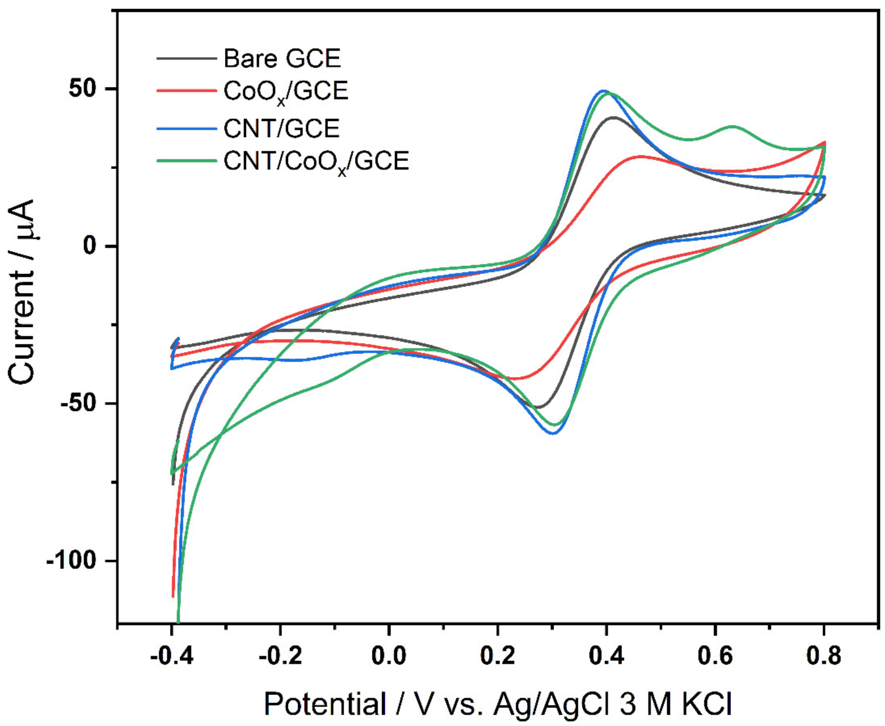

Figure S2. Cyclic voltammograms of the GCE with different modifications in 5 mM $K_3[Fe(CN)6]$ containing 0.1 M KCl at the scan rate of 50 mV/s.

Table S2. Comparison of the electroactive surface area for the electrode with different modifications estimated from CV in 5 mM $K_3[Fe(CN)_6]$ containing 0.1 M KCl.

Electrode	Electroactive Surface Area (cm^2)
Bare GCE	0.0580
CoO_x/GCE	0.0345
SWCNT/GCE	0.0682
CoO_x/SWCNT/GCE	0.0637

Table S3. Effect of interfering substances on the current response for BHC determination (50 μM) using the CoO_x/SWCNT/GCE.

Substance	Concentration (mM)	%Deviation
NH_4Cl	5	−1.43
$MgCl_2$	5	−3.21
KNO_3	5	−4.16
glucose	5	−0.42
sucrose	5	−2.72
ribose	5	−5.43
caffeine	5	−6.20
